# Calcium ATPase (PMCA) and GLUT-4 Upregulation in the Transverse Tubule Membrane of Skeletal Muscle from a Rat Model of Chronic Heart Failure

**DOI:** 10.3390/ijms252011180

**Published:** 2024-10-17

**Authors:** Sofia Gitler, Ibrahim Ramirez-Soto, Aura Jiménez-Graduño, Alicia Ortega

**Affiliations:** 1 Department of Biochemistry, School of Medicine, Universidad Nacional Autónoma de México, Mexico City 04510, Mexico; i3ramire@uwaterloo.ca (I.R.-S.); aura.jimenez@udlap.mx (A.J.-G.); 2 Department of Internal Medicine, ABC Medical Center, Sur 136 166, Alvaro Obregón, Mexico City 01120, Mexico; 3 Department of Health Sciences, Universidad de Las Américas Puebla, San Andrés Cholula 72810, Mexico

**Keywords:** transverse tubule membrane, chronic heart failure, skeletal muscle, PMCA, GLUT4, sarcoplasmic reticulum

## Abstract

Intolerance to exercise is a symptom associated with chronic heart failure (CHF) resulting in SM waste and weakness in humans. The effect of CHF on skeletal muscle (SM) arose from experimental evidence in rat models to explain the underlying mechanism. We investigated SM mechanical and metabolic properties in sham rats and with coronary ligation-induced CHF. After twelve weeks of CHF, rats were catheterized to measure right auricular pressure, SM mechanical properties, SERCA-ATPase activity and plasma membrane Ca^2+^-ATPase (PMCA) hydrolytic activity in isolated sarcoplasmic reticulum (SR) and transverse tubule (TT membrane), respectively, in the sham and CHF. The right auricular pressure and plasma nitrite concentration in CHF increased two-fold with respect to the sham. Pleural effusion and ascites were detected in CHF, confirming CHF. SERCA activity was conserved in CHF. In TT membranes from CHF, the glucose transporter GLUT4 increased seven-fold, and the PMCA hydrolytic activity increased five-fold, but in isolated muscle, the mechanical properties were unaffected. The absence of a deleterious effect of coronary ligation-induced CHF in the rat model on SM could be explained by the increased activity of PMCA and increased presence of GLUT-4 on the TT membrane, which may be involved in the mechanical outcome of the EDL.

## 1. Introduction

Skeletal muscle (SM) weakness, atrophy and limited exercise capacity are common symptoms that classically coincide with the development of chronic heart failure (CHF) in patients [1]. Rat models of CHF have been used to study whether SM functionality is directly related to CHF in humans. The hypothesis of increased susceptibility to muscle fatigue in CHF patients has been raised from experimental evidence in rat models that has been divisive in several instances [2,3,4]. There is evidence that shows a decrease in tension during twitch and tetanic stimulation [2,5], but other studies have found no changes in either fast or slow SM using the same CHF model [3,4]. In studies related to Ca^2+^ regulation, the data have shown either a decline [2,6], an increase [7] or no changes in Ca^2+^ transients [8,9] in isolated SM from CHF rats. Reduced blood flow (BF) has been commonly proposed as the main mechanism of muscle dysfunction [10]; however, there is also direct evidence for normal muscle tissue perfusion during CHF [11]. After improving cardiac performance in CHF rats [11,12,13] and in patients [14,15], there is no parallel improvement from SM when exercise tolerance or muscle activity is evaluated. Furthermore, the most consistent finding in CHF patients and coronary ligation-induced CHF rat models is the increase in the plasma nitric oxide (NO) concentration [16,17]. Nitric oxide synthase (NOS) activity in SM resulted in a significant increase of NOS-I and NOS-II in CHF rats [18] and in humans [19], indicating the importance of NO production not only in the vascular endothelium but within SM.

During differentiation, the plasma membrane of the myotube folds into the cell-forming caveolae, which leads to the formation of an extended transcellular web of surface membranes, named the transverse tubule (TT) system [20], which creeps into the muscle fibre, increasing the total surface area to be around seven times more than Sarcolemma [21], which is in contact to the extracellular environment. Two events are associated with the development of the TT system: (1) the transmission of the action potential to initiate excitation/contraction coupling initiated by the Dihydropyridine Receptor, a voltage-dependent calcium channel, and the transfer of the insulin-mediated glucose transporters to the surface through the glucose transporter type 4 (GLUT4), an insulin-dependent GLUT isoform expressed in SM and adipose tissue [22], both being the key markers of TT membranes in adult SM. GLUT4 is the main mechanism for SM glucose uptake in response to insulin and exercise in adult life, and it has been found to be enriched three-fold in TT membranes compared to the sarcolemma in adult SM [23]. In gestational undernourished animals, GLUT4 is overexpressed and muscle force is higher in the undernourished when compared to the normal rats [24,25,26]. The occurrence of GLUT4 overexpression represents a mechanism of SM adaptation.

In patients with CHF and SM waste and atrophy, the IGF-1 levels are reduced [27] and can increase after exercise training [28]. Although muscle waste and reduced muscle force have not been seen in all cases of CHF in the rat model [29,30], in general, exercised individuals are less predisposed to suffer muscle cell damage [31]. The physiological compensatory response to endurance exercise, known as adaptation, may be the overexpression of GLUT4 [24], among other factors where the consequence is a prolonged enhancement of one of the Ca^2+^ homeostatic regulatory mechanisms, thereby maintain resting [Ca^2+^]_i_ at or below 1 × 10^−7^ M. If the SERCA1 activity is enhanced, more Ca^2+^ would be transported into the SR; however, SERCA1′s capacity has a limit. It has been shown that, during repetitive tetanic activation, the TT lumen Na^+^ concentration ([Na^+^]_TT_) decreases, whereas the intracellular Na^+^ concentration ([Na^+^]_i_) increases, the Na^+^ concentration reduces the Na electrochemical gradient (∆μNa) and, thereby, the Na^+^/Ca^2+^ exchange activity decreases, leading to an increase in [Ca^2+^]_i_ [32]. Therefore, sustained muscle activity induces a major adaptive response of the muscle cell by upregulating other Ca^2+^ regulatory mechanisms, such as the TT membrane Ca^2+^-ATPase (PMCA), which represents approximately 10% of the total ATPase activity in TT membranes in adult SM [33], which has been shown to be overexpressed after a protocol of endurance exercise in rabbits [34].

This study was undertaken to examine whether there are changes in the mechanical properties—force, fatigue and fatigue recovery—in fast SM isolated from a coronary ligation-induced CHF rat model. The SR function and TT membrane Ca^2+^-ATPase and GLUT4 expression in CHF were determined with respect to the sham animals.

## 2. Results

### 2.1. Myocardial Infarction and Parameters to Determine CHF

**Figure 1:**
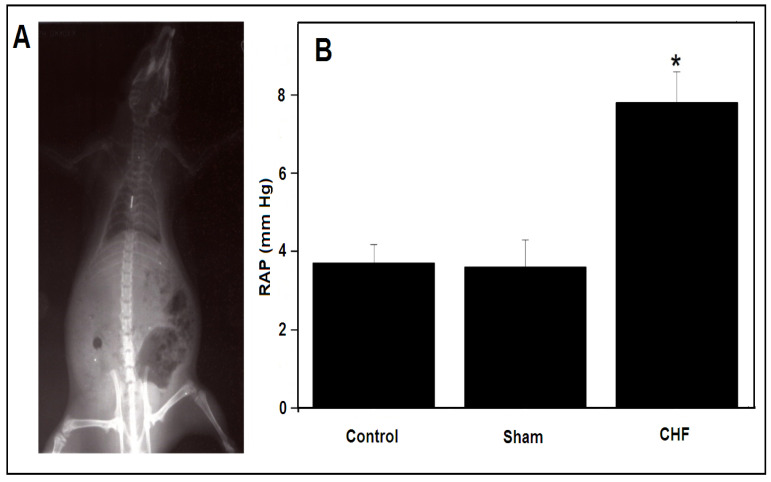
Right auricular blood pressure (RAP) determination. (**A**) A barium fluoroscopy image, where the catheter tip was localized to confirm the correct RAP. (**B**) RAP of the animals in the control, sham and CHF groups (±SDM, control *n =* 10, sham *n =* 10 and CHF *n =* 10). * *p* < 0.05.

The mortality rate in the animals subjected to CAL was 12%, compared to 8% subjected to the sham procedures. Twelve weeks after the surgical procedure, the physiological characteristics of the CHF animals were compared to those of the sham animals. Electrocardiographic patterns such as ST-segment elevation, deep T-wave inversion, the formation of abnormal Q-waves, the presence of a right bundle branch block and ventricular extrasystoles were frequently observed. The myocardial infarcts area had extensions of 21% to 43% in the CHF animals, while no infarctions were observed in the sham group. Figure 1A shows a fluoroscopic image used to evaluate the catheter localization for right auricular pressure (RAP) determination. Figure 1B shows the differences in the RAP between the groups. The control animals (with no surgery) had a RAP of 3.7 ± 0.48 mmHg, the sham group had a RAP of 3.6 ± 0.69 mmHg and the CHF group had a RAP of 7.8 ± 0.78 mm Hg (±SD, *n =* 10). Table 1 summarizes the morphometric and hemodynamic characteristics of the CHF and sham animals. Pleural effusion and ascites were the only abnormal conditions detected in the CHF rats.

The high RAP observed in CHF rats was an indication of a chronic condition. We determined that the presence of NO confirmed a compromised vasculature under these conditions.

### 2.2. Plasma Concentration of Nitric Oxide

**Figure 2:**
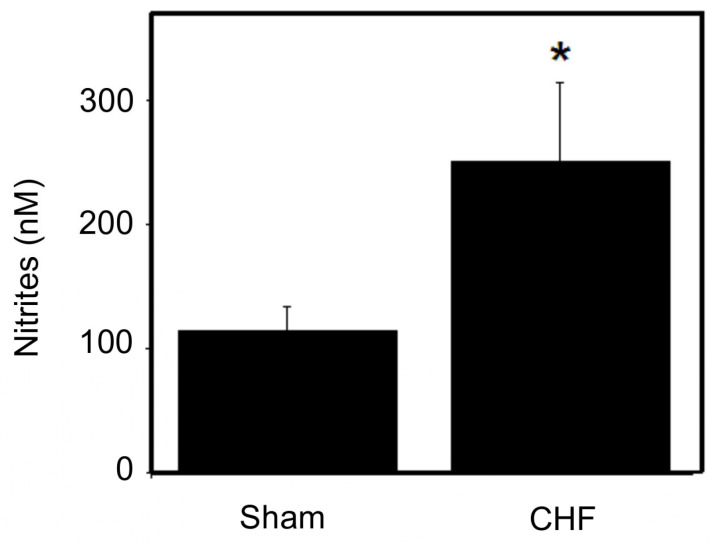
Plasma nitrite concentration. Sham: 115.08 ± 18.63 nM (7.7 ± 0.6 μg); CHF: 251.78 ± 63.33 nM (17.4 ± 2.1 μg) (±SDM, sham *n =* 4 and CHF *n =* 4). * *p* < 0.05.

Figure 2 shows the plasma nitrite concentration, which was 2.2 times higher in the CHF rats. The plasma nitrite concentration was 115.08 ± 18.63 nM (7.7 ± 0.6 μg) (±SD, *n =* 4) in the sham rats and 251.78 ± 63.33 nM (17.4 ± 2.1 μg) (±SD, *n =* 4) in the CHF rats. Because the plasma NO concentration was higher in CHF rats, we examined the systemic effect of NO on SM function through the mechanical properties.

### 2.3. SERCA1 ATP Hydrolytic Activity and ATP-Dependent Ca^2+^ Uptake in the LSR

**Figure 3:**
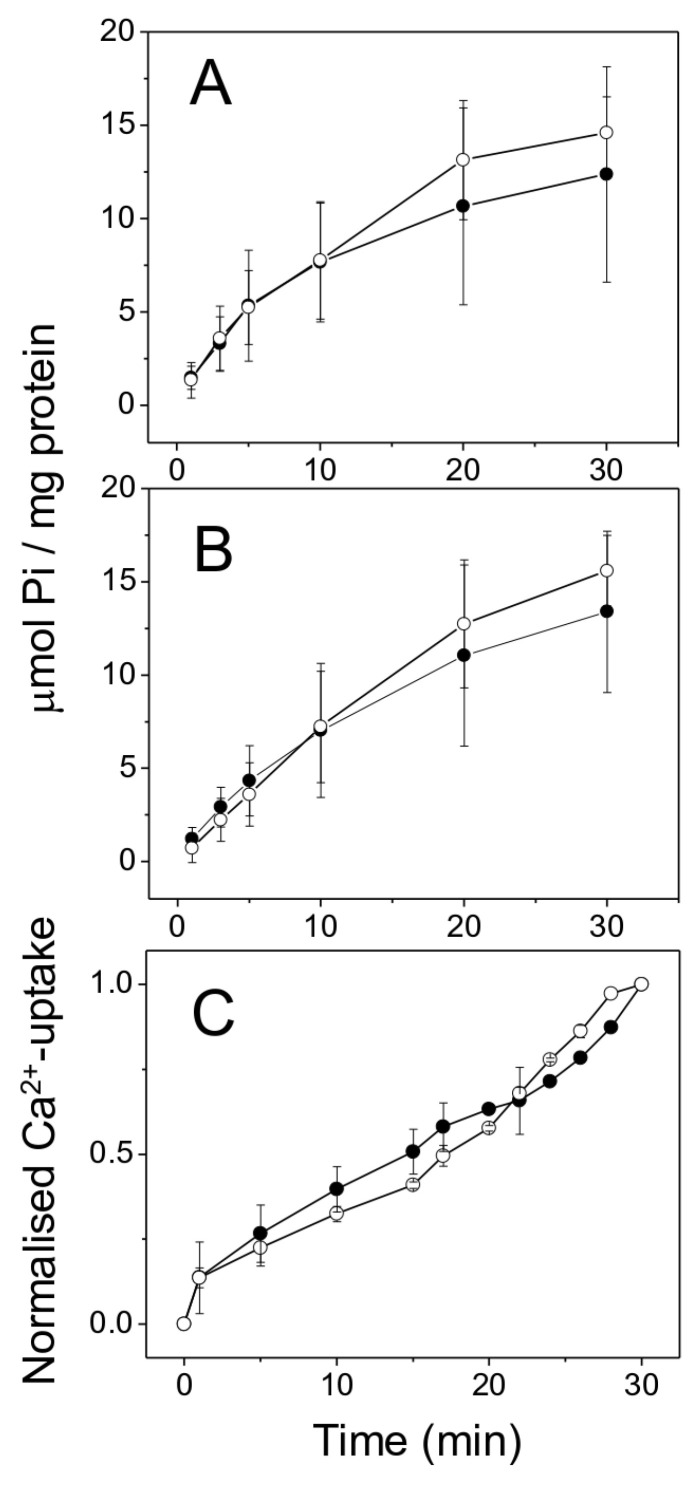
SERCA1 ATP hydrolytic activity and calcium uptake in isolated SR. (**A**) ATP hydrolytic activity in the sham group (±SDM, *n =* 3) in the absence (solid symbols) or presence of 1 mM BAL (open symbols). (**B**) ATP hydrolytic activity in the CHF group (±SDM, *n =* 3) in the absence (solid symbols) or presence of 1 mM BAL (open symbols). (**C**) ATP-dependent Ca^2+^ uptake in the presence of 5 mM potassium oxalate. Sham, solid symbols; CHF, open symbols (±SDM, sham *n =* 4 and CHF *n =* 4). *p* < 0.05.

Figure 3A shows the ATP hydrolytic activity of SERCA1 in the sham group (12.38 ± 5.76 μmol P_i_/mg protein) with no effect in the presence of 1 mM BAL (14.6 ± 1.94 μmol P_i_/mg protein) (±SD, *n =* 3). Figure 3B shows the ATP hydrolytic activity of SERCA1 in the CHF group (13.4 ± 4.32 μmol P_i_/mg protein), with no effect in the presence of 1 mM BAL (15.59 ± 1.89 μmol P_i_/mg protein, ±SD, *n =* 3). Therefore, there were no significant differences in SERCA1 hydrolytic activity between the groups. To obtain further evidence for preserved SERCA1 activity, we measured Ca^2+^ uptake in isolated SR. Figure 3C shows that there were no differences in ATP-dependent Ca^2+^ uptake between the sham and CHF groups. The Ca^2+^ uptake activities measured after 30 min were 200.1 ± 22.6 nmol ^45^Ca/mg protein in the sham group and 211.4 ± 4 nmol ^45^Ca/mg protein in the CHF group (±SD, *n =* 10).

### 2.4. Transverse Tubule Membrane Mg^2+^-ATPase and PMCA Hydrolytic Activity

**Figure 4:**
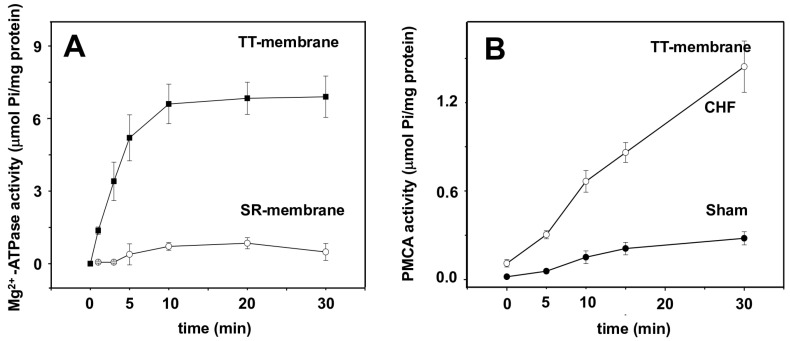
ATPase hydrolytic activity of the TT membrane and sarcoplasmic reticulum. (**A**) Mg^2+^-ATP hydrolytic activity in isolated TT membranes (solid symbols) and in SR membranes from CHF’s rats in the presence of 1 mM EGTA (open symbols) (±SDM, *n =* 3). (**B**) PMCA activity in the sham group (solid symbols) and in the CHF (open symbols) (±SDM, *n =* 6).

Figure 4A shows the Mg^2+^-ATP hydrolytic activity in CHF-isolated TT and SR membranes. In the absence of Ca^2+^, SERCA is inactive (0.48 ± 0.34 μmol P_i_/mg protein), while in the TT membrane, the most abundant TT membrane ATPase is fully active (6.9 ± 0.85 μmol P_i_/mg protein) (±SD, *n =* 3). Figure 4B shows the PMCA hydrolytic activity in isolated TT membranes from the sham group at 30 min of reaction (0.28 ± 0.04 μmol P_i_/mg protein), and in the TT membrane isolated from CHF, the ATP hydrolytic activity of PMCA increased (1.44 ± 0.17 μmol P_i_/mg protein) (±SD, *n =* 6) when compared to the sham group. The increment of specific ATPase activity was five times more in TT membranes isolated from CHF SM.

### 2.5. Glut4 Expression in Transverse Tubule Membranes

**Figure 5:**
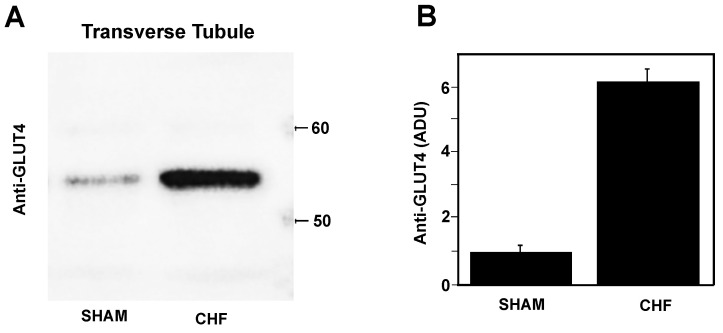
GLUT4 in TT membranes. (**A**) A representative anti-Glut4 Western blot on isolated TT membranes from the sham and CHF skeletal muscle. (**B**) The statistical representation in arbitrary density units (ADU). Normalized units; sham (1) and CHF (6.7 ± 0.25) (±SDM, *n =* 3).

Figure 5A shows a representative immunoblot for anti-GLUT-4 in isolated TT membranes from the sham and CHF fast SM. Figure 5B shows the normalised densitometry units (NDUs) with respect to the α-actin loading reference for GLUT4 membrane expression demonstrated a significant seven-fold increase (6.7 ± 0.25 NDU) in CHF compared to the sham 1 (0.95 ± 0.2 NDU).

### 2.6. Mechanical Properties of Fast Skeletal Muscle

**Figure 6:**
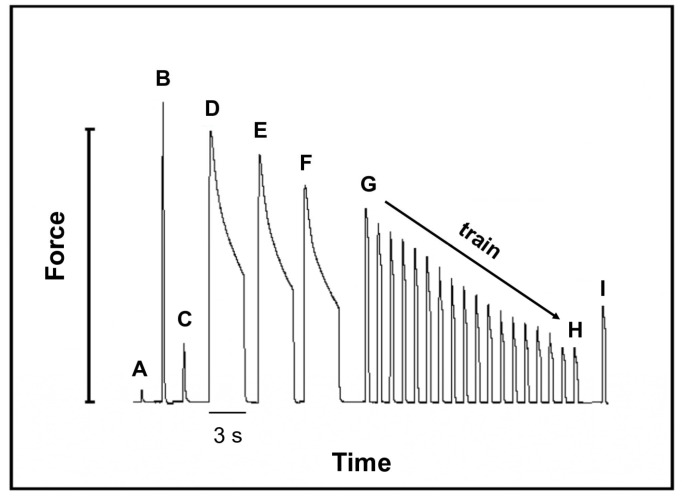
Mechanical protocol used in isolated EDL muscle. (**A**) Single twitch (1 Hz, 1 V). (**B**) Tetanus (75 Hz, 100 V, 1 s). (**C**) Single twitch (1 Hz, 100 V). (**D**–**F**) Fatigue protocol; three tetanic stimulations of 3 s at intervals of 3 min (75 Hz for 3 s, 100 V). (**G**,**H**) Fatigue train protocol (75 Hz, 100 V, repeated every 3 s to reach 30% of the initial force). (**I**) Force recovery after fatigue; tetanus (75 Hz, 100 V, 1 s) after 5 min of rest.

Figure 6 shows a diagram of the protocol used for the analysis of the mechanical characteristics where the letters are associated with the specific force development associated with the stimulation procedure (described under “Experimental Procedures” Section 4.7).

Table 2 lists the results of this protocol for the sham and CHF. We observed no differences between the low- and high-voltage stimulations. Figure 6 shows the protocol, where peak (A) corresponds to the forces obtained at the optimal muscle length: 0.11 ± 0.032 N and 0.135 ± 0.042 N for the sham and CHF groups, respectively. Peak (B) corresponds to the maximum forces induced by tetanic stimulation; 1.358 N and 1.363 N for the sham and CHF groups, respectively. Peaks (D to F) correspond to the residual force (given as a percentage of the maximal force of each tetanic contraction) was calculated from the difference between the maximum and minimum forces). The average for the three stimulations was 65 ± 15% in the sham group and 74 ± 14% in the CHF group, indicating that there were no significant differences between the groups. Difference between peaks (G-H) is the time required to achieve a 70% decrease in muscle force during the tetanic training and was the same for the sham and CHF groups (66 ± 10.1 s and 68.2 ± 7.6 s, respectively). At the end of the fatigue protocol, the recovery forces peak (I) were 54 ± 8.4% and 52.9 ± 10.3% in the sham and CHF groups, respectively, for all the measurements (±SD, *n =* 10).

## 3. Discussion

For several years, the relationship between SM weakness and CHF observed in patients has been studied. In our study, we found that, in our rat model of CHF, (1) changes in RAP are a direct indicator for CHF; (2) the [NO] in the vascular bloodstream is increased two-fold; (3) there is no mechanical failure in isolated fast SM, preserving force development, the time course of fatigue development and force recovery after fatigue; (4) the ATP hydrolytic activity of SERCA1 and ATP-dependent Ca^2+^ transport are unaffected for up to 12 weeks of CHF; (5) the PMCA hydrolytic activity increases by five-fold in TT membranes from CHF rat SM; and (6) TT membrane’s GLUT4 content increases by six-fold in SM from CHF rats.

### 3.1. RAP vs. Left Ventricular End-Diastolic Pressure (LVEDP) to Determine CHF

CAL in rats has been the preferred technique used to study the pathophysiology of SM once CHF is settled. To determine the status of CHF, the maximum rate of left ventricular pressure increase (LV *d*P/*d*t_max_) has been used as a traditional criterion for determining the unremitting cardiac damage associated with CHF. However, left ventricular end-diastolic pressure (LVEDP) is a parameter that is related to contractile dysfunction after massive myocardial infarction (MI), which does not necessarily resolve in CHF.

In 2001, Lunde et al. reviewed in detail the use of LVEPD measurements in rats with CAL and questioned the reliability of this parameter for identifying CHF [7]. The proposal of using echocardiographic criteria to correlate the heart damage associated with CHF revealed the disadvantages in LVEDP determination [35]. In the present study, we used the determination of RAP as direct evidence for venous blood return to the heart, because the right auricular pressure is the last parameter to be affected before the cardiac output is modified. Consequently, this condition is considered necessary to trigger the impairment of the peripheral tissues. Cardiac output and venous blood return are directly correlated; therefore, any change in the cardiac output is a result of the changes in the factors that determine the venous blood return to the heart. It is known that MI does not necessarily result in a high RAP, but the outcome of a high RAP is always related to CHF. In our experience, RAP is a direct indicator of CHF in rats.

### 3.2. [NO] in the Vascular Bloodstream

One of the principal compensatory mechanisms in CHF is an increase in the plasma [NO], which is an important factor for vasodilatation [36]. Hambrecht et al. demonstrated that there is an increase in NOS-I in SM biopsies from patients with CHF, indicating a potential participation of NO in the regulation of blood flow (BF) [37,38]. Systemic vasoconstriction is elevated in CHF via sympathetic nervous efferent activity, which is due to impaired NO-mediated vasodilatation in patients [37]. However, increased NOS activity has been found in cardiac tissue, SM and the blood during CHF. Rush et al. observed that CAL-induced CHF in rats, NOS-I and NOS-II is overexpressed in SM [39]. Although it is known that NO affects BF, substantial experimental evidence directly related to SM function indicates that [NO] has no effect on the mechanical properties of muscle. Didion et al. demonstrated that, several weeks post-infarction, arteriolar reactivity is impaired after chronic MI, demonstrating that the arteriolar resistance of rat spin trapezius muscle is reduced after CHF, likely because of a decrease in NO production [40]. Therefore, this finding favours the hypothesis that reduced BF is the cause for SM damage during CHF in rats. Musch et al. used radioactive microsphere (15 µm in diameter) techniques for CAL-induced MI and measured BF in several organs and diverse slow and fast SMs in the sham and infarcted animals during rest and after exercise [10]. They found no changes in BF in all organs tested and observed reduced BF in almost all SMs; however, there was no difference between the resting and post-exercise groups. Schiǿtz et al. used the same microsphere technique in rat CHF models and showed that the BF in the soleus exhibited no reduction in BF when compared to the sham [11]. Bernocci et al. used a different rat model for CHF (monocrotaline) and determined (using fluorescent microspheres) that the BF in neither the soleus nor the EDL is decreased at rest [16].

Because we found a significant increase in the plasma [NO] in CHF rats and no change in muscle mechanical properties, upholding BF may be one the protective mechanisms within the SM to overcome the effect of CHF through the increment in [NO].

### 3.3. SERCA1 Activity Is Not Affected by CHF

Cunha et al. proposed that exercise prevents oxidative stress and proteosome activity reverting SM atrophy caused by HF [41]. Because of its redox potential, SERCA1 has been described as a member of the family of vicinal thiol proteins and is inhibited by the selective oxidation of adjacent cysteine disulphide bonds, which depend on the intracellular redox potential [42]. Our results on SERCA1 when in the presence of the selective reducing agent BAL (Figure 4B) does not have a significant effect of SERCA1 activity, demonstrating that no oxidative damage was observed after 12 weeks of CHF.

The fact that the plasma and intracellular [NO] are increased during CHF could suggest an inhibitory effect on SERCA, since this enzyme is redox regulated [42]. However, our results demonstrate that neither hydrolytic activity nor ATP-dependent Ca^2+^ uptake is affected by CHF for twelve weeks. Williams and Ward showed that the force production of the gastrocnemius muscle induced by a wide range of stimulation frequencies was not different in a group of rats with CAL-induced myocardial infarction [43]. However, they showed that the ATP-induced SR Ca^2+^ release and ATP-dependent Ca^2+^ uptake were significantly affected. Since we did not observe a significant difference in SR Ca^2+^ uptake, we interpreted that the increase in Ca^2+^ uptake and release previously described [43] suggest faster Ca^2+^ movement in and out of the myofilament space, which may be corelated to why these authors observed no effect on SM force production, as we also did not observe mechanical changes in the EDL in this work.

### 3.4. Mg^2+^-ATPase Activity Marker of TT Membranes Is Not Present in SR

The Ca^2+^/Mg^2+^-ATPase known as Mg^2+^-ATPase is primarily found in TT membranes [33,44], and the Mg^2+^-ATPase can be activated either by [Mg^2+^] in the absence of calcium or by mM [Ca^2+^] acting as a marker of a TT membrane during isolation. In SR, the Mg^2+^-ATPase is absent; instead, SERCA is the primary ATPase activity, which is dependent on Mg^2+^ and substantially activated by nM [Ca^2+^]. Consequently, the isolated TT membrane used in this work is a highly enriched fraction.

### 3.5. Ca^2+^-ATPase (PMCA) Activity in TT Membranes Is Affected by CHF

Another homeostatic mechanism that could extrude the extra cytosolic Ca^2+^ that might be accumulated during prolonged muscle activity is the Na^+^/Ca^2+^ exchanger located in the Sarcolemma [45]. The exchanger is driven by the Na^+^ electrochemical gradient (∆μ_Na_) at normal [Na^+^]_TT_ of 120 mM; however, [Na^+^]_TT_ is reduced to ~60 mM during a 60-Hz, 2- to 3-s tetanus [32]. The 60 mM [Na^+^]_TT_ moves into the cytosol and increases intracellularly [Na^+^]_i_. The [Na^+^]_TT_ depletion and [Na^+^]_i_ increase cause a substantial decrease in ∆μ_Na_. The decrement in ∆μ_Na_ in the resting membrane potential that occurs during high-frequency activation led to low activity of the exchanger, resulting in an increased cytosolic [Ca^2+^] [32]. The SR may take up the extra Ca^2+^ not extruded by the Na^+^/Ca^2+^ exchanger. However, the frequency of stimulation does not allow for a complete Ca^2+^ removal by the SR as the rate of Ca^2+^ uptake decreases. These combined factors will lead to an increase in cytosolic [Ca^2+^]_i_ that alters excitation-contraction coupling, contractility and, subsequently, viability of the muscle cells [46]. Thus, another mechanism must be activated to reduce the increased long-term cytosolic [Ca^2+^]_i_. The results presented in this study show an increase in TT membrane PMCA activity. Thereby, we propose that during CAL- induced myocardial infarction and CHF in the rat model, where SERCA seems to be unaffected, the six-fold increase in PMCA hydrolytic activity indicates that SM cytosolic [Ca^2+^]_I_ in CHF could be higher due to an undergoing systemic stress experienced by the increase in NO production.

### 3.6. TT Membrane GLUT4 Content in SM of CHF Rats

The specific activity of the TT membrane PMCA was higher in CHF, which may be considered as an adaptative mechanism of the SM to a chronic condition. Although some other studies using the same protocol of CAL induces myocardial infarction in the rat have shown muscle waste and loss of force after 12 weeks of CHF, our study showed that the morphometric and physiological parameters were conserved after 12 weeks of chronic condition. The increase in TT membrane GLUT4 expression found in this study supports the hypothesis of a compensatory mechanism in nutrient-stressed animals during a chronic condition. However, it is necessary to further investigate the structural functionality of GLUT4, which is overexpressed in the TT membranes of the CHF in rats. Issa V. et al. in 2010 showed that CHF in patients often occurs simultaneously with diabetes, and the prognostic of the patient depends on glycemia [38]. Under systemic stress conditions, SM can react with either an increase in isometric or isotonic contraction where glucose consumption is needed; therefore, the insulin demand is higher and would explain the increase in GLUT4 observed.

### 3.7. Mechanical Function in Fast SM Is Preserved after CHF

Studies regarding the failure of SM activity in patients with CHF have been directed toward raising evidence for the impact of CHF on SM. Several experimental approaches have provided evidence that points in various directions. This is where the paradigm is present. Perrault et al., using the same model for CHF, found reductions in the twitch force, tetanic force, relaxation rate and Ca^2+^ signal decay six weeks post-infarction and concluded that CHF had a significant effect on the mechanical properties of the EDL muscle in this rat model, suggesting a potential mechanism that explained the effect of CHF on SM function in patients [2]. However, in 2002, Lunde et al. found no differences in the maximal force, contraction rate and relaxation rate between the CHF and sham groups six weeks post-infarction in a rat CHF model with a perfused EDL [29].

In the soleus and diaphragm muscles, tetanic tension in rats with CAL-induced CHF compared to sham rats 12 to 14 weeks post-infarction decreased [4]. However, substantial evidence has provided different results. Arnolda et al., in 1991, demonstrated (in rat gastrocnemius, plantaris and soleus muscles stimulated via the sciatic nerve) that there are no differences in the maximal forces of CAL-induced CHF and sham animals [3]. Okada et al., in 2008, found in vastus lateralis muscle biopsies no significant differences in the unloaded shortening velocities and the maximal forces of CHF patients and controls, concluding that SM contractile protein function is preserved in human heart failure [30]. Therefore, any change in muscle activity, if any, is unrelated to the contractile elements. Rehn et al., in 2009, showed that, in CAL-induced CHF in rats, the maximal force development, contraction rate and relaxation rate of each stimulation train sixteen weeks post-infarction are not significantly different between the CHF and sham groups. However, temporary fatigue was observed after six weeks, with a significant difference in the contraction and the relaxation rates but no difference in the maximal force [4]. Although the authors interpreted these results in support of an effect of CHF on SM, in this CAL model, the lack of differences in the mechanical properties of the EDL in our study of twelve weeks post-infarction included no effect.

Even though an increase [NO] does not interfere with the function of contractile proteins, its increment in plasma may affect the regulation of intracellular calcium in SM through the increase in TT membrane PMCA activity and GLUT4 expression, which could contribute to explain the conservation of SM function in the rat CHF model. Further investigation of the effect of a high plasma [NO] on SM is needed to propose a mechanism describing the metabolic state and calcium regulation of SM in the rat model of CHF. We conclude that the CAL-induced CHF rat model, provides interesting results to understand the physiology of SM, yet is insufficient to explain the failure of SM in the pathophysiology of human CHF.

## 4. Materials and Methods

All procedures were conducted in accordance with the Guide for the Care and Use of Laboratory Animals of the Institute of Laboratory Animal Resources of the United States, as approved in Mexico by the Ethics Committee of the School of Medicine of the National Autonomous University of Mexico (UNAM) (NOM-062-ZOO1999) [47]. 

### 4.1. Animals

Male Wistar rats three months old (250–300 g) were used for surgery. The rats used in this study were obtained from the Bioterio Academic Unit, Faculty of Medicine, Universidad Nacional Autónoma de México. When specified, they were euthanized by cervical dislocation after being anesthetized in a CO_2_ chamber. At room temperature (25 °C), the EDL muscles were dissected for mechanical experiments and the rest of fast skeletal muscle was used for membrane isolation (sarcoplasmic reticulum and transverse tubule membranes).

### 4.2. Surgical Protocol for Left Anterior Descending Artery Occlusion and Measurement of Right Auricular Pressure (RAP)

Two experimental groups were used. The animals in the experimental group (CHF group), in which the left anterior descending coronary artery was ligated (CAL), as described previously [35,48]. Briefly, under anaesthesia, 87 mg/kg of 2-(2-chlorophenyl)-2-(methylamino)cyclohexan-1-one (ketamine) and 13 mg/kg of 2-(2-chlorophenyl)-2-(methylamino)cyclohexan-1-one (xylazine) (PISA Pharmaceutics, Mexico City, Mexico) and artificial ventilation (1 mL O_2_/100 g wt) (683 Rodent Ventilator, Harvard Apparatus, Holliston, MA, USA), a thoracotomy and a left anterior descending artery ligation were performed under a microsurgical microscope (OP-MI, Carl Zeiss, Oberkochen, Germany) (*n =* 10). The animals in the second group underwent a sham surgery, in which the coronary artery was exposed but not occluded (sham group) (*n =* 10). Ketamine is a N-methyl-d-aspartate receptor antagonist that produces anaesthesia and analgesia with minimal depression of the cardiovascular system. Xylazine is an α2 adrenergic receptor agonist that causes sedation and muscle relaxation and has analgesic properties. The combination of ketamine/xylazine has been a frequent choice for mouse/rat anaesthesia and was the protocol used for previous research done for the rat CHF studies [2]. The advantages are the convenience of a single injection, results for an easier animal handling, well-controlled anaesthetic depth and duration and fewer side effects after being awoken. We used 83 mg/kg of 2-acetyloxybenzoic acid, 2,6-diaminohexanoic acid (Vetalgina) (IntervetShering, Plough Animal Health, Boxmeer, The Netherlands) an antipyretic, analgesic, antispasmodic and anti-inflammatory pharmaceutical for post-surgery recovery. After surgical recovery, the animals were kept under observation in a temperature-controlled room with a 12/12-h light/dark cycle for twelve weeks with free access to food and water. The right auricular blood pressure (RAP) was measured using a pressure transducer and 3-Fr catheter (MAI-72, Harvard Apparatus, MS, USA) placed into the right auricle. Fluoroscopies were performed to follow the barium-labelled catheter, as observed in Figure 1.

### 4.3. Electrocardiography Trace

Nine weeks after surgery, an electrocardiogram was recorded (MP-100 system, Biopac Systems, Fremont, CA, USA) in the sham group and in the rats with CAL. A lead DI was used for all the traces, where the DI is the voltage between the left (positive) and right (negative) arm electrodes.

### 4.4. Myocardial Infarct Size Measurement

After pressure transduction, the hearts were dissected into the left ventricle, right ventricle and inter-ventricular septum and weighed. The infarct size was measured using triphenyl tetrazolium chloride [49] (Sigma-Aldrich, St. Louis, MO, USA). Heart slices were incubated in a buffer containing NaH_2_PO_4_ (0.1 M) with a pH of 7.4 and 1% *w/v* tetrazolium salt for 20 min at 37 °C.

### 4.5. Nitrite Determination

Blood samples (4 mL) were obtained from the inferior vena cava. Plasma nitrites were measured using the Griess reagent (Invitrogen by Thermo Fisher Scientific, Waltham, MA, USA). The nitrates were reduced to nitrites via exposure to nitrate reductase [50] (*E. coli* 1775, ATCC, Manassas, VA, USA) (*n =* 4 sham and *n =* 4 CHF).

### 4.6. Tissue Sampling

At the appropriate times, the heart, liver and EDL muscles were dissected and weighed. Other parameters relevant to CHF, such as pleural effusion and ascites, were measured (*n =* 10 sham and *n =* 10 CHF).

### 4.7. Experimental Procedure for Muscle Force Recording

The *extensor digitorum longus* (EDL) was isolated at room temperature. The isolated muscle was placed into an acrylic chamber that was equipped with platinum electrodes along each side of the chamber wall to allow contact with the Krebs solution containing (in mM): 135 NaCl, 5 KCl, 1 MgCl_2_, 2.5 CaCl_2_, 11 dextrose, 1 NaPO_4_ dibasic and 15 NaHCO_3_ and a gas mixture of 95% O_2_ and 5% CO_2_ to reach a pH of 7.0. The EDL muscle was fastened by its distal tendon to a forceps and by its proximal tendon to a force transducer (FT-03, Grass Medical Instruments, West Warwick, RI, USA). The platinum electrodes were connected in parallel to two stimulators (SD9, Grass Medical Instruments, RI, USA), as previously described [51,52] (10 sham and *n =* 10 CHF).

### 4.8. Mechanical Protocol

The muscles were stretched to the optimum length at which the twitch force was maximal (1 Hz, 1 V and 100 V), and the muscles were at their optimum sarcomere length of 2.5 µm; see the protocol in Figure 6 (peaks A,C). A tetanic stimulation of 75 Hz for 1.0 s at 100 V was performed before the fatigue protocol to establish the maximum force, Figure 6 (peak B). The fatigue protocol was as follows: A tetanic EDL stimulation of 75 Hz for 3 s and 100 V was performed three times, with a resting period of 3 min between stimulations, Figure 6 (peaks D–F). Before the fatigue training protocol began, the EDL was at rest for 5 min, and a training stimulation of 75 Hz for 1 s at 100 V was repeated every 3 s to reach 30% Figure 6 of the initial force of the training, Figure 6 (peaks G-H). After the training protocol, the muscle rested for 5 min, and a new stimulation of 75 Hz at 100 V for 1 s was performed to observe fatigue recovery, Figure 6 (peak I) [39,40,51,52]. The muscle force expressed in Newtons (N) was determined using a mass calibration curve (*n =* 10 sham and *n =* 10 CHF).

### 4.9. Isolation of T-Tubules and SR Membranes

Membranes were obtained from the fast SM (hind limbs and back muscles) of the sham and CHF rats. The membranes were isolated using differential centrifugation and a discontinuous sucrose gradient, as previously described [33,53]. Membrane isolation was performed in the absence of any reducing agents in the buffer medium. The microsomal fraction was placed into a sucrose gradient of 25%, 27.5%, 35% and 43% *w*/*v*. The 25%/27.5% interface had the maximal signal for the dihydropyridine receptors, as determined by immunoblotting, indicating that this interface corresponded to the TT membrane. Light SR (LSR) membranes were isolated from the 27.5%/35% interface, which was detected by the maximum ATPase activity stimulated by Ca^2+^. Protein concentrations were determined using the CoomassiePlus Protein Assay Reagent (Pierce, Rockford, IL, USA), with BSA as the standard (*n =* 3 sham and *n =* 3 CHF).

### 4.10. Immunoblotting

TT membrane proteins were separated by SDS-PAGE on a 10% polyacrylamide gel and transferred to nitrocellulose membrane. Membranes for Western blot were blocked at least 30 min with 5% non-fat dry milk (Bio-Rad, Hercules, CA, USA) and incubated with the anti-GLUT-4 (ab62375-100) and an anti-α-actin loading control (A1801), all obtained from Abcam (Waltham, MA, USA) primary antibody. After washing and incubation with the corresponding peroxidase-labelled secondary antibody (Invitrogen) for 45 min, blots were developed and densitometric analysis was performed with ImageJ v.1.54 (*n =* 3 sham and *n =* 3 CHF).

### 4.11. SERCA1 and PMCA Hydrolytic Activity

ATPase activity was determined by the colorimetric determination of phosphate using malachite green, as described previously [54]. For SERCA, aliquots of 5 μg/mL of protein SR were incubated in a solution containing (in mM) 100 KCl, 5 MgCl_2_, 5 NaN_3_, 0.1 CaCl_2_, 0.33 ATP and 20 Tris-malate pH 7.0 in the presence or absence of 1 mM BAL (British anti-Lewisite, 2-3-dimercaptopropanolol) at a pH of 7.0. For PMCA and Ca^2+^/Mg^2+^-ATPase (total ATPase), aliquots of 10 μg/mL of protein TT membrane were incubated in a solution containing (in mM) 100 KCl, 5 MgCl_2_, 5 NaN_3_, 0.1 CaCl_2_, 0.33 ATP and 20 tris-malate pH 7.0 in the absence and in the presence of 1 EGTA for PMCA and Ca^2+^/Mg^2+^-ATPase, respectively, as previously described [33,55]. In all cases, the reaction was stopped using a solution containing 0.045% malachite green hydrochloride, 4.2% ammonium molybdate in 4 N HCl, 0.8 mL Triton X-100 per 100 mL of solution and 0.25 mL Na_3_C_3_H_5_O(COO)_3_ (34%) (Sigma Aldrich, St. Louis, MO, USA). The absorbance was read at 660 nm. These experiments were performed on isolated LSR from the fast SM of the sham and CHF rats (*n =* 3 sham and *n =* 3 CHF).

### 4.12. Calcium Uptake

Calcium transport was measured by the filtration method at room temperature (25 °C) in a solution containing (in mM) 100 KCl, 5 MgCl_2_, 0.1 CaCl_2_, 5 K_2_C_2_O_4_, 1 μCi[^45^CaCl_2_], 20 Tris-malate (pH 7.0), and 4 ATP (Sigma Aldrich, St. Louis, MO, USA), with 0.6 mg of protein/mL. The reaction was stopped using 0.5 mL of ice-cold quenching solution containing (in mM) 5 MgCl_2_, 4 EGTA and 20 Tris-malate (pH 7.0) (Sigma Aldrich, MO, USA). Membrane vesicles (0.9 mL) were filtered through 0.45-μm filters (Merck Millipore, Burlington, MA, USA), washed, dried and then counted via scintillation. These experiments were performed on isolated SR from the muscles of sham and CHF rats (*n =* 3 sham and *n =* 3 CHF).

### 4.13. Statistical Analysis

All the values are expressed as mean ± standard deviation. The parameters of the sham and CHF groups were compared using the two-tailed unpaired Student *t*-test. *p*-values of <0.05 were statistically significant. Statistical analysis was performed using OriginPro 8 SR0 (OriginLab Corporation, Northampton, MA, USA).

## Figures and Tables

**Table 1:** Morphometric and hemodynamic variations between the sham and CHF groups.

	Sham	CHF
Initial body wt (g)	277.5 ± 9.2	262.6 ± 15.8
Final body wt (FBW) (g)	442 ± 45.8	458.1 ± 45
*RAP (mm Hg)*	*3.6 ± 0.7*	7.8 ± 0.8 *
Liver wt (mg)	16686 ± 3043	15910 ± 2902
Heart wt (mg)	1375 ± 221	1376 ± 150
LV wt (mg)	595.6 ± 105	596.3 ± 82
RV wt (mg)	436.3 ± 66	381.8 ± 71
Septum wt (mg)	313.8 ± 70	296.8 ± 44
Liver/FBW (mg/g)	37.8 ± 6.1	34.62 ± 6.47
Heart/FBW (mg/g)	3.1 ± 0.31	3.05 ± 0.63
LV/FBW (mg/g)	1.31 ± 0.21	1.32 ± 0.3
RV/FBW (mg/g)	0.96 ± 0.13	0.84 ± 0.18
Septum/FBW (mg/g)	0.69 ± 0.14	0.65 ± 0.14
*Pleural effusion (μL)*	*0*	125 ± 35.4 *
*Ascites (μL)*	*0*	171.7 ± 20.3 *

FBW, final body weight; RAP, right auricular blood pressure; LV, left ventricle; RV, right ventricle (±SDM, sham *n =* 10 and CHF *n =* 10). * *p* < 0.05.

**Table 2:** Comparative parameters obtained from the force recorded from the mechanical activity of the EDL muscle in the sham and CHF groups. Maximum force (Max F); minimum force (Min F) (±SDM, sham *n =* 10 and CHF *n =* 10). * *p* < 0.05.

Extensor Digitorum Longus	Sham	CHF
[*A*] Twitch, optimum muscle length (1 V) (N)	0.110 ± 0.032	0.135 ± 0.042
[*B*] Tetanus, maximum force (N)	1.358 ± 0.301	1.363 ± 0.338
[*C*] Twitch (100 V) (N)	0.101 ± 0.022	0.134 ± 0.035 *
[*D*] First tetanus, maximum force (N)	1.261 ± 0.302	1.320 ± 0.403
[*D*] First tetanus, minimum force (N)	0.902 ± 0.373	1.036 ± 0.433
[*D*] ∆ First tetanus (Max F − Min F) − residual force (%)	69.8 ± 16.3	75.5 ± 15
[*E*] Second tetanus, maximum force (N)	1.099 ± 0.257	1.173 ± 0.387
[*E*] Second tetanus, minimum force (N)	0.711 ± 0.261	0.920 ± 0.404
[*E*] ∆ Second tetanus (Max F − Min F) − residual force (%)	63.4 ± 12.3	75.5 ± 12.8
[*F*] Third tetanus, maximum force (N)	0.909 ± 0.243	1.038 ± 0.370
[*F*] Third tetanus, minimum force (N)	0.591 ± 0.320	0.768 ± 0.383
[*F*] ∆ Third tetanus (Max F − Min F) − residual force (%)	61.8 ± 17.5	70.8 ± 14.1
[*G*] Train, tetanus (Max F: 100%) (N)	0.793 ± 0.238	0.948 ± 0.287
[*H*] Train, tetanus at 30% respect to Max F (N)	0.24 ± 0.07	0.285 ± 0.091
[*H*] Time to reach 30% of the initial force in train protocol (development of fatigue) (s)	66 ± 10.1	68.2 ± 7.6
[*I*] Recovery tetanus − 5 min (%)	54 ± 8.4	52.9 ± 10.3

*n =* 10 Sham and *n =* 10 CHF; N = Newtons.

## Data Availability

Data contained within the article.
